# Sharing the Same Perspective. Mental Disorders and Central Serous Chorioretinopathy: A Systematic Review of Evidence from 2010 to 2020

**DOI:** 10.3390/biomedicines9081067

**Published:** 2021-08-23

**Authors:** Gianluca Pandolfo, Giovanni Genovese, Antonio Bruno, Diletta Palumbo, Umberto Poli, Sebastiano Gangemi, Pasquale Aragona, Alessandro Meduri

**Affiliations:** 1 Department of Biomedical and Dental Sciences, Morphological and Functional Images, University of Messina, 98121 Messina, Italy; gpandolfo@unime.it (G.P.); antonio.bruno@unime.it (A.B.); diletta.palumbo.dot@gmail.com (D.P.); umberto_poli@hotmail.it (U.P.); pasquale.aragona@unime.it (P.A.); alessandro.meduri@unime.it (A.M.); 2 School and Operative Unit of Allergy and Clinical Immunology, Policlinico “G. Martino”, Department of Clinical and Experimental Medicine, University of Messina, 98125 Messina, Italy; gangemis@unime.it

**Keywords:** central serous chorioretinopathy, mental disorders, insomnia, neuroinflammation, psychiatry

## Abstract

Background: The relevance of the association between mental disorders and other conditions might have been underestimated due to its complexity. Central Serous Chorioretinopathy (CSC) is an ophthalmological disorder associated with many psychiatric factors. The aim of this systematic review is to evaluate the association between mental disorders and CSC. Methods: Articles about studies performed on humans on CSC published in peer-reviewed journals from 1 January 2010 to 31 December 2020 were included in the review. Results: We selected 21 research papers. Nine studies measured stress and anxious depressive symptoms, which are associated with CSC onset and recurrences, emerging as a state marker of the disease. Four out of the five studies focused on sleep disorders suggested a reliable association with CSC. Four studies evaluated other various psychiatric factors. The role of psychopharmacological medication has still not been elucidated (three studies). Conclusion: Multiple pieces of evidence highlights that CSC might arise in the context of systemic disease. This notion, together with the increasing evidence supporting a link between psychiatric disorders and choroidal thickness, suggests that CSC and mental disorders may share some etiopathogenetic pathways. Further research is needed to better investigate possible common etiopathogenetic pathways, especially vascular, immunological and endocrinological systems.

## 1. Introduction

Mental disorders (MD) are common diseases associated with an increased risk of developing other medical conditions, higher service utilization and a great impact on health care costs [1,2]. Acknowledged associations are with cardiovascular, immunological and neurological diseases and, although explanatory models have still not been elucidated, a shared etiology has been suggested [3,4]. However, the relevance of the association between MD and other conditions might have been underestimated due to its complexity and proteiform manifestation [5]. In this context, the association between Central Serous Chorioretinopathy (CSC) and MD could be of great interest, as it might be helpful to investigate the etiopathogenesis of different psychiatric and ophthalmological diseases.

CSC is a common ophthalmological disorder characterized by serous detachment of the neurosensory retina in association with thickening and hyperpermeability of the choroid [6]. Symptoms of CSC include central scotoma, micropsia, metamorphopsia, defective color vision and difficulty in-depth perception. Known risk factors are mid-age, male sex, smoking, hypertension and H. Pylori infection [7]. Acute CSC patients recover spontaneously within 3 to 6 months, so conservative treatment is often recommended. Recurrent episodes may be noted in about 50% of untreated cases while, in a smaller percentage, CSC can evolve to a chronic disease, with increased risk of permanent visual loss due to persistent neurosensory retinal detachment and retinal pigment epithelium (RPE) atrophy [8]. CSC is associated with exposure to increased levels of endogenous or exogenous glucocorticoids. Indeed, different studies have suggested that CSC pathogenesis is strongly related to hypothalamic–pituitary–adrenal (HPA) axis dysregulation, which is also involved in stress response and in various psychiatric conditions [9]. Several studies have associated CSC with many psychiatric factors like depressive symptoms, anxiety, stress, and sleep disorders [10,11,12]. The use of psychopharmacological therapy has also been found more frequently in patients with chronic CSC [13].

The aim of this review is: (1) to evaluate the current state of research to investigate the association between MD and CSC patients; (2) to investigate whether MD influences the risk of chronicization for CSC patients.

## 2. Materials and Methods

### 2.1. Search Processes

This systematic review was conducted according to PRISMA (Preferred Reporting Items for Systematic Reviews and Meta-Analyses) guidelines [14]. First, the search strategy was developed and completed in PubMed, and then the same strategy was applied to Scopus. Databases were searched until 31 December 2020, using the following key terms: “Central serous chorioretinopathy”, “Mental disorder”,” anxiety”, “sleep disorder”, “insomnia”, “depression”, “panic”, “psychiatry”. The electronic search strategy used for PubMed is described in Table 1. Additional articles were researched by detecting similar articles and articles with titles containing our search terms. Articles were selected by title and abstract; the entire article was read if abstract concerned psychiatric features in CSC patients. Further investigations were carried out on the references of the articles that emerged.

### 2.2. Study Selection

**Figure 1:**
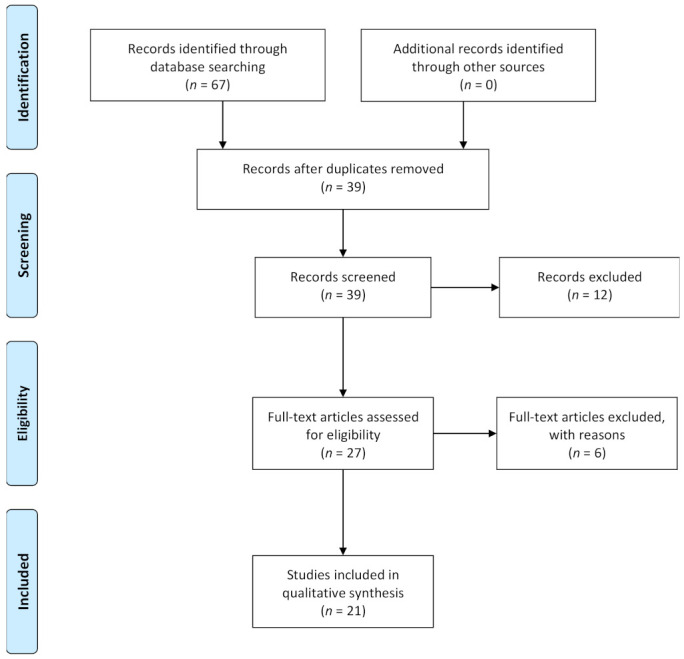
Flow diagram of the literature selection process.

Articles were included in the review according to the following inclusion criteria: English OR Italian language, publication in peer-reviewed journals from 1 January 2010 to 31 December 2020, articles about studies performed on humans on CSC. Articles were excluded by title, abstract or full text for irrelevance to the topic in question. Further exclusion criteria were articles published before 1 January 2010, not written in English OR Italian language, unpublished dissertations and theses, and other non-peer-reviewed material. Figure 1 shows the diagram of the literature selection process.

### 2.3. Data Extraction

Two authors (GP, GG) performed the initial search, independently reviewed and selected the references based on the inclusion and exclusion criteria. The results were subsequently re-evaluated by the auditors and the salient results were shown. Data derived from our research of articles included study author names, publication dates, study designs (i.e., open-label uncontrolled and randomized controlled trial), sample (case and control group), tests used for the assessment, and main findings.

## 3. Results

According to the literature, we found 21 research papers that evaluated psychiatric factors in CSC patients. The results are reported for major topics and summarized in chronological order in Table 2. Nine studies measured stress and anxious depressive symptoms, five studies focused on sleep disorders, four studies evaluated other various psychiatric factors, and three studies described the impact of psychopharmacological medication. With regard to CSC subtypes, we found six manuscripts reporting data about acute CSC, four studies evaluated chronicization in CSC, and four studies investigated recurrence in CSC.

We found four studies using data from the Taiwan National Health Insurance Research (TNHIR) database [11,16,29,31]. Tien et al. (2020) made a longitudinal retrospective cohort study to investigate the association between non-organic sleep disturbance (NOSD) and CSC. Data include 53.743 NOSD patients without CSC compared to fourfold (214.972) random controls without sleep disturbance or CSC. They found that NOSD patients have a higher incidence of CSC than controls independently of any other comorbidity. Male patients with a history of steroid usage within 1 year before CSC development were at higher risk to develop CSC than women and non-steroid usage. The subgroup using sleeping pills had a higher risk of CSC compared to subjects not using drugs for insomnia [16]. Tsai et al. (2013) evaluated risk factors for CSC unrelated to corticosteroid use with a retrospective cohort study. Data from 786 CSC patients (500 males) and 3606 (2294 male) controls were analyzed. Exposure to anti-anxiety drugs within a one-year period before enrolment was independently associated with idiopathic CSC among males only [31]. Chen et al. (2019) studied risk for depression in 25,939 CSC patients in a retrospective cohort study. They found that the CSC group had a significantly higher risk for depression [11]. Data from (TNHIR) were investigated also by Chang et al. (2015) with a case-control study design. A large sample of 2921 CSC patients and 17,526 controls was analyzed. Patients with CSC had a significantly higher prevalence of psychiatric comorbidity [29]. We found another longitudinal cohort study by Nicholson et al. (2018). They retrospectively investigated electronic records about clinical characteristics associated with visual outcomes in 258 CSC patients from a single center. The use of psychiatric medication at presentation was protective for vision loss, and there was a trend toward a protective effect with the use of beta-blockers (known for anxiolytic effect) [22].

Numerous other pieces of evidence emerged about psychiatric conditions and CSC in transversal studies. Ji et al. (2018) investigated sleep quality and CSC (both acute and chronic disease) in a sample of 134 treatment-naive patients compared to 134 HC. Poor sleep quality (The Pittsburgh Sleep Quality Index > 5) and stress were significantly more frequent in CSC groups than in HC. CSC patients showed more frequent relevant depression and anxiety symptoms than controls [23]. Penas et al. (2020) investigated cerebrovascular regulation and anxiety in 20 CSC patients (both with active or inactive disease) and 14 HC with a case-control study design. The State-Trait Anxiety Inventory (STAI) score was significantly higher in CSC patients compared to controls. Among CSC patients 25% showed a high level of anxiety-state and a 55%-high level of anxiety-trait according to the cut-off of the STAI. They found that both active and inactive CSC patients had impaired Neurovascular Coupling (NVC) mechanisms, which leads to altered cerebrovascular regulation. NVC was calculated after the N-Back Task, describing changes in cerebral mean blood flow velocity before and after an executive task. Moreover, NVC was negatively associated with STAI scores, especially anxiety-state scores [15]. In contrast, Kim YK et al. (2018) investigated psychological dimensions and CSC phases and subtypes (active and inactive disease, among active CSC they distinguish between acute and chronic). The sample includes 37 CSC patients and 37 HC. In the inactive CSC groups, the results did not differ in all psychological dimensions tested compared with HC, including STAI and The Beck Depression Inventory (BDI), the latter evaluating depression symptoms [21].

Balkarli et al. (2017) investigated fibromyalgia (Fm) frequency in a sample of 83 CSC patients with active symptoms, compared with 201 HC. The presence of a known psychiatric disorder was among the exclusion criteria. In the CSC group, patients with Fm showed higher scores in BDI and BAI scales than patients without Fm. Results in BDI and BAI scales did not differ between patients with CSC and Fm compared with Fm controls [26]. The same group investigated sexual function in 58 CSC patients without known psychiatric disorders compared to 99 HC. CSC patients showed a higher incidence of erectile dysfunction than controls. Sexual desire score, evaluated with the 15-question Index of Erectile Function-15 (IIEF-15), was negatively associated with CSC duration. No BDI and BAI results were reported [25]. Uhumwangho et al. (2015) described a cases series of five CSC patients. All patients had a positive history of underlying anxiety or stressful conditions [28].

### 3.1. Acute CSC

Sahin et al. (2014) examined 30 patients affected by acute CSC and 30 HC. CSC group showed significantly higher scores than controls in all symptomatic dimensions at Symptom Checklist 90-R (SCL-90-R) (Somatization *p* = 0.002; Obsessive–compulsive *p* = 0.007; Interpersonal sensitivity *p* = 0.002; Depression *p* = 0.005; Anxiety *p* = 0.003; Anger-hostility *p* = 0.015; Phobic anxiety *p* = 0.009; Paranoid ideation *p* = 0.004; Psychoticism *p* = 0.016) and lower score at all subscale Short Form-36 assessing quality of life (Physical functioning *p* = 0.032; Physical role difficulty *p* = 0.012; General health perception *p* = 0.032; Vitality *p* < 0.001; Social functioning *p* = 0.001; Emotional role difficulty *p* = 0.013; Mental health *p* = 0.013) except Bodily pain (*p* = 0.079) [30]. Scarinci et al. (2020) investigated psychological profile and cortisol production in 14 subjects affected by severe obstructive sleep apnea (OSA) without ophthalmological disease, 14 matched HC without sleep disorders, 14 newly diagnosed CSC patients with acute symptoms, and 14 matched HC without ocular abnormalities. The use of chronic medication was an exclusion criterion. CSC subjects showed significantly higher scores than controls at the Daily Hassles and Stress scale (DHS) and at the Hamilton Rating Scale for Depression (HAM-D), resulting in a medium level of perceived stress and borderline mean level of depressive symptoms. No anxiety was reported at The Hamilton Anxiety Rating Scale (HAM-A) [17]. The same research team analyzed dysregulation of stress response-modulating systems and psychological profiles in a sample of 17 patients suffering from an initial acute episode of CSC and 17 HC. Chronic CSC, recurrent CSC and use of vasoactive drugs were exclusion criteria. They found significantly higher scores in CSC acute patients than control on the negative subscale of The Positive and Negative Affect Schedule (PANAS), on the DHS, and on The Beck Depression Inventory-II (BDI-II). The results at the BDI-II were driven by the scores on depressive–somatic effective in contrast to cognitive items. No significant differences in the STAI scores were found between the two groups [18]. Other results about acute CSC emerged from the above-mentioned studies. The cohort study from Chen et al. (2019) found that the risk for depression in the CSC group was higher at onset compared with recurrent episodes [11]. In accordance with this study, Kim YK et al. (2018) found that acute CSC, but not chronic CSC, is associated with depression symptoms assessed by BDI, the severity of depression showed a linear correlation with the size of the choroidal lesion in this group [21]. Moreover, sexual satisfaction was higher in acute CSC than chronic CSC subgroup [25].

### 3.2. Chronic CSC

Chronic CSC, but not acute CSC, was associated with significantly higher stress levels than controls [21]. Yu et al. (2019) investigated risk factors for CSC chronicization, they examined patients who had spontaneous resolution of the disease after the first episode and no recurrence within 1 year, compared to patients affected by persistent or recurrent CSC (20 First episode CSC, 118 chronic CSC). Male sex and older age were correlated to chronic CSC. Patients with chronic CSC had a significantly higher level of Insomnia severity index (ISI) [19]. Buosquet et al. (2016) investigated the association between sleep disturbance and CSC in a sample of 40 CSC patients. They found that CSC patients had significantly more sleep disturbance than HC, especially in chronic compared with acute subtypes. The two groups did not differ regarding history of depression [12]. Setrouk et al. (2016) investigated alteration in circadian rhythm in 29 patients affected by chronic CSC compared to 29 patients with other ocular conditions. There were no significant differences between the two groups in sleeping, depressive and anxiety disorders (assessed by The Hospital Anxiety and Depression scale), or psychopharmacologic medication use [27].

### 3.3. Recurrent CSC

A retrospective case series from Matet et al. (2018) investigated candidate risk factors for recurrent CSC in 46 patients with acute CSC. History of depression, psychological stress, and sleep disorder were not associated with recurrences of CSC [24]. Bazzazi et al. (2015) investigated anxiety associated with the occurrence of CSC in 30 patients at the first or second episode of disease. Anxiety scores, evaluated with The Hamilton Anxiety Rating Scale (HAM-A), were higher in CSC patients compared with healthy control (HC), independently from sex and number of episodes [10]. Fok et al. (2011) investigated risk factors for recurrence in 73 CSC patients without treatment with 3 years of follow-up. Patients with a history of psychiatric illness were associated with an increased risk of CSC recurrence [32]. Jain (2019) reported a case of CSC associated with the use of Quetiapine (200 mg) in a 30-year-old male with insomnia. After the withdrawal of medication, he showed a marked reduction in both signs and symptoms of CSC. He showed recurrence of the disease when resumed quetiapine 10 months later [20].

## 4. Discussion

Patients with acute CSC show a significantly higher prevalence of psychiatric comorbidity in a very large case-control study [29]. Increasing evidence correlates the CSC with anxiety [10,15,23,28,30] and stress [21,23,28]. High levels of anxiety were found in 30 CSC patients, both at first episode and relapses [10]. One study investigating chronic CSC in 29 patients failed to find a significantly high level of anxiety-depressive symptoms [27]. Various studies showed an association between CSC and depression especially during acute episodes [11,17,18,21,30]. A study based on a large sample from Chen et al. (2019) reported that patients at the onset of CSC showed a higher risk for depression, which decreased after the resolution of the first CSC episode and did not modify after receiving ophthalmological treatment [11]. Caution must be taken in interpreting the other results based on a small sample. Chronic CSC patients seem not to differ from HC about depressive symptoms [27]. Remarkably, the correlation with anxious–depressive condition was related to the active phase of disease, while it was not detected during inactive phase assessment [21], and it was not associated with recurrence of disease [24]. On combining this result, stress, anxiety and depression levels emerged as state markers of CSC, while inactive CSC patients reported no differences in psychological factors compared with HC. In contrast, two other studies from the same group reported no difference in anxiety scores between very small samples of CSC patients at onset and HC [17,18], these studies has an important risk of bias: patients with chronic pharmacological therapy [17] or vasoactive therapy [18] were excluded from the sample. In fact, high evidence levels from longitudinal cohort studies correlate exposure to sleeping pills [16] and anti-anxiety drug [31] with a higher risk of CSC. Both studies [16,31] were performed based on the TNHIR database. However, this association could be due to the presence of a mental disorder itself, rather than the psychopharmacological treatment, which has even been proposed as a protective factor for vision outcome in CSC patients at onset [22]. Still, the Quetiapine assumption was correlated to exacerbation of CSC in a case-report study [20].

All the studies focusing on CSC and sleep disorders, except one [27], suggested a reliable association between the diseases, as patients with CSC had a higher rate of insomnia or poor sleep quality than HC [12,23], and patients with non-organic sleep disturbances showed an increased incidence of CSC than controls independently of any other comorbidity [16]. Moreover, one study highlighted insomnia as the only modifiable risk factor for persistent or recurrent CSC, so treatment for sleep disturbances was strongly recommended [19]. Otherwise, evidence about other psychiatric and/or psychological factors and their association with recurrences of CSC are contrasting [24,32].

It is known that psychological stress, through the activation of the HPA axis, stimulates cortisol release, which is an established risk factor for CSC [9]. Furthermore, this link between stress and steroids seems to regard particularly male patients, who showed a positive correlation between cortisol levels and neuroticism, while females showed a negative correlation. This evidence may partly explain the wide gender gap in CSC prevalence [33]. However, it is still not entirely clear how corticosteroids, despite having anti-inflammatory properties, can worsen, rather than improve, the course of a disease characterized by inflammation. Researchers suggested the possibility of tissue-specific or conditional mutation of steroid hormone receptors in the choroid [34]. It is hypothesized that the pathogenesis of CSC extends beyond a tissue-specific disorder, as its comorbidity with psychological stress, hypertension and coronary diseases may suggest the involvement of systemic vascular dysfunction. In this regard, anxiety and cerebrovascular regulation were evaluated in 20 CSC patients and 14 HC, results showed that CSC patients had a significant impairment of NVC, which was also negatively associated with anxiety levels, especially in state scores, both in active and inactive CSC patients [15]. Another possible pathophysiological mechanism underlying this association is inflammation. To date, few studies investigated the role of inflammatory mediators in CSC. A promising result emerged from a recent study that highlighted IL-6 dysregulation in 60 CSC patients, which might be correlated to elated cortisol level and vascular permeability during stress response [35].

Recent evidence supports a link between psychiatric disorders and choroidal thickness, regardless of the presence of CSC or another ophthalmological diagnosis: Ayyildiz D and Ayyildiz T (2020) [36] reported a significant thickening of the central choroid in 41 children and adolescents with anxiety disorders compared with HC. Likewise, further evidence indicated that choroidal thickness is significantly increased in adult patients with primary insomnia [37], bruxism [38] and obsessive–compulsive disorder [39]. Regarding insomnia, previous authors suggested that excessive exposure to light, through the increase in retinal dopamine and the subsequent nitric oxide release, may lead to an upper blood flow in the choroid and, ultimately, to its thickening [40]. Finally, one study reported that choroidal thickness was higher in 100 patients with major depressive disorder (MDD) than controls, especially at first episode than recurrent MDD patients [41]. Moreover, in a sample of 37 CSC patients, choroidal lesion size showed a linear correlation with the severity of depression [21]. These results are particularly interesting since they suggest that a link between psychiatric conditions and choroid alterations may be detected even without any ophthalmological symptom. In this context, the hypothetical role of choroidal thickness as a state marker of psychiatric disorders needs further investigations.

As mentioned above, an association between anxiety and choroidal thickening was found also in children and adolescents. Nonetheless, within this age group, the relationship between psychiatric and ophthalmological factors does not extend to CSC, as reports of childhood CSC cases are very rare [34]. Anxiety, together with the coping strategies to deal with it, mainly involve serotoninergic and dopaminergic neural circuits starting from the prefrontal cortex, which reaches full maturation at approximately 25 years of age [42]. Researchers, assuming that the neural elements of stress response play a crucial part in CSC pathogenesis, proposed this maturation lack of the prefrontal cortex as the reason why the disease does not occur in childhood and adolescence [34]. However, the role of monoamine metabolism in the development of CSC is still to be clarified.

## 5. Conclusions

The retina is part of the central nervous system and is subject to physiological modification in relation to mental status and emotion. This regulation can be altered by abnormal stimulation, exogenous factors, and innate predisposition, leading to specific syndromes as CSC or specific alteration in the context of mental disorders. The strongest evidence comes from studies on sleep disorders, which appear to be actively involved in the pathophysiology of CSC and its chronicization. Anxiety and stress disorders appear to be related to the active phases of the disease, while patients at the onset of CSC showed a higher risk for depression, which decreased after the resolution of the first CSC episode. In our previous work, we hypothesized that maladaptive personality traits might represent a possible link between psychiatric symptoms, CSC, and endocrinological patterns [43]. This review has broadened our perspective. CSC might arise in the context of a systemic disease, correlated to vascular, immunological and endocrinological systems. The evidence that retinal alterations were found in patients with MD, together with the evidence that patients with CSC showed higher levels of psychiatric symptoms, suggests that CSC and MD may share some etiopathogenetic pathways which might be also associated with alterations in the monoamine metabolism or a common neuroinflammatory mechanism. Thus, CSC could be a useful explanatory model to investigate high psychiatric comorbidity in stress correlated disease. Further research is needed to better understand the bidirectional influences from psychiatric and psychological features and vision mechanisms.

## Figures and Tables

**Table 1:** List of search terms entered into databases search engines in order to identify the articles.

Number	Search Term
1	“Central serous chorioretinopathy”
2	“Mental disorder”
3	“anxiety”
4	“sleep disorder”
5	“insomnia”
6	“depression”
7	“panic”
8	“psychiatry”
9	1 AND 2 OR 3 OR 4 OR 5 OR 6 OR 7 OR 8 OR 9 OR 10
10	English OR Italian [language]
11	up to 31 December 2020 [publication date]

**Table 2:** Selected manuscripts evaluating psychiatric factors in Central serous chorioretinopathy (CSC) patients.

Reference	Study Design	Aim	Subjects	Assessment	Main Findings
[15]	Case-control study	To investigate cerebrovascular regulation and anxiety in Central Serous Chorioretinopathy (CSC).	20 CSC patients (acute, chronic, inactive) and 14 healthy controls (HC)	State-Trait Anxiety Inventory (STAI)	Neurovascular Coupling was negatively associated with STAI scores, especially anxiety-state scores (r = −0.602, *p* = 0.002).
[16]	Retrospective cohort study	to investigate nonorganic sleep disturbance (NOSD) and CSC.	53.743 NOSD patients without CSC, 214.972 random controls.	/	NOSD patients have a higher incidence of CSC than controls (HR, 1.65; 95% CI, 1.34–2.02). In the NOSD groups, subjects using sleeping pills had an even higher CSC risk (1.72-fold, 95% CI, 1.35–2.20).
[17]	Case-control study	To investigate obstructive sleep apnea (OSA) and CSC.	14 OSA patients and 14 matched HC; 14 acute CSC patients and 14 matched HC.	Hamilton Rating Scale for Depression (HAM-D); The Hamilton Anxiety Rating Scale (HAM-A), and the Daily Hassles Scale (DHS)	CSC subjects showed a medium level of perceived stress (scores between 76 and 115 on the DHS scale), HDS and HAM-D scores were significantly higher than controls (*p* < 0.001; *p* < 0.05); no anxiety was reported.
[18]	Cross-sectional observational study	To investigate stress, psychological profile, and CSC.	17 initial acute CSC patients and 17 HC.	The Positive and Negative Affect Schedule (PANAS); STAI; The Beck Depression Inventory-II (BDI-II); DHS.	CSC group scores were higher than control on the negative subscale of the PANAS (*p* = 0.023), the DHS (*p* < 0.001), and the BDI-II (*p* = 0.036). No significant differences in the STAI scores were found.
[19]	Cross sectional	To investigate risk factors for chronic CSC.	20 First episode CSC, 118 chronic CSC (46 persistent CSC; 72 recurrent CSC).	Insomnia Severity Index (ISI)	Higher Insomnia Severity Index score is associated with persistent or recurrent CSC (*p* = 0.015).
[11]	Retrospective cohort study	To investigate the risk for depression in CSC patients.	25,939 CSC patients (first onset and recurrent) and 103,756 controls.	/	CSC group had a significantly higher risk for depression (*p* < 0.0001; HR = 1.29); treatment of CSC did not significantly reduce the risk for depression (HR = 0.85).
[20]	Case report	To describe Quetiapine administration and possible related CSC.	A 30-year-old male with insomnia	/	After the withdrawal of medication, he showed a marked reduction in both signs and symptoms of CSC. He showed recurrence of disease when resumed quetiapine 10 months later.
[21]	Case-control study	To investigate psychological dimensions and CSC phases and subtypes	9 chronic CSC and 10 acute CSC (active CSC phase), 18 inactive CSC phase, and 37 HC	STAI; BDI; Social Readjustment Rating Scale; the Coping Inventory for Stressful Situations; Medical Outcomes Study Social Support Survey.	Inactive CSC groups results did not differ in all psychological dimensions compared with HC. Acute CSC patients reported higher scores in depression (*p* = 0.029), the severity of depression showed linear correlation with the choroidal pathology (R2 = 0.622; *p* = 0.007). Chronic active CSC was associated with stress level (*p* = 0.024).
[22]	Retrospective cohort study	To investigate clinical characteristics and visual outcomes in CSC.	258 CSC patients	/	Use of psychiatric medication at presentation was protective for vision loss (*p* = 0.0066). There was a trend toward a protective effect with the use of beta-blockers, known for the anxiolytic effect (*p* = 0.060).
[23]	Case-control study	To investigate sleep quality and CSC.	134 treatment-naive CSC patients and 134 HC	Depression Anxiety Stress Scales 21-item version (DASS-21); The Epworth Sleepiness Scale (ESS); The Pittsburgh Sleep Quality Index (PSQI)	Poor sleep quality (PSQI > 5) and stress (*p* < 15) was significantly more frequent in CSC groups than in HC (*p* < 0.0001; *p* = 0.002). CSC patients showed more frequent relevant depression (*p* > 10) and anxiety (*p* > 8) symptoms than controls (*p* = 0.001; *p* = 0.008).
[24]	Retrospective Case series	To investigate risk factors for recurrent CSC.	46 patients with acute CSC	/	History of depression, psychological stress, and sleep disorder was not associated with recurrences of CSC.
[25]	Case-control Study	To investigate CSC and erectile dysfunction (ED).	58 patients with CSC (12 acute, 46 chronic), 99 HC.	BDI; BAI; The 15-question Index of Erectile Function-15 (IIEF-15).	CSC patients showed higher incidence of erectile dysfunction than controls (*p* < 0.001). Sexual desire score at IIEF-15 was negatively associated with CSC duration (*p* = 0.025). Sexual satisfaction was higher in acute CSC than chronic CSC group (*p* = 0.016).
[26]	Case-control Study	To investigate fibromyalgia (Fm) among CSC patients.	83 patients with CSC (23 acute, 58 chronic), and 201 HC.	BAI; BDI.	In the CSC group, patients with Fm showed higher scores in BDI and BAI scales than patients without Fm (*p* < 0.001).
[27]	Case-control study	To investigate circadian rhythm and CSC.	29 chronic CSC; 29 patients non-CSC.	The PSQI; The Epworth questionnaire; The Hospital Anxiety and Depression scale	There were no significant differences between the two groups in sleeping, depressive and anxiety disorders, or psychopharmacologic medication use.
[12]	Case-control study	To investigate sleep disturbances and CSC.	40 active CSC (26 acute; 14 chronic) and 40 HC	ISI.	CSC patients had significantly more sleep disturbance than HC (*p* < 0.001), especially in chronic than in the acute subtype (*p* < 0.05). There was no significant difference between the two groups about depression.
[28]	Descriptive case series	To investigate clinical presentation in CSC.	5 CSC	/	All patients had a positive history of underlying anxiety/stressful conditions.
[29]	Case-control study	To investigate corticosteroid use and CSC.	2.921 acute CSC; 17.526 controls	/	Patients with CSC had a significantly higher prevalence of psychiatric disease (*p* < 0.001).
[10]	Case control study	To investigate anxiety associated with the occurrence of CSC.	17 CSC at first episode (8 female, 9 male), 13 CSC at second episode (5 female, 8 male); 30 HC.	HAM-A	Anxiety scores were significantly higher in CSC patients compared with HC (*p*-value not reported), independently from sex and number of episodes. No statistically significant correlations were observed between the anxiety scores and choroidal alteration and duration of the CSC episode
[30]	Case-control study	To investigate Quality of Life in CSC patients	30 acute CSC patients, 30 HC.	the Symptom Checklist 90-R (SCL-90R), Short Form-36 (SF-36)	CSC group showed higher scores than controls in all symptomatic dimensions at SCL-90-R (global severity index *p* = 0.001) and lower score at all subscale SF-36 except bodily pain. They found a negative correlation between depression subscale scores and visual acuity (r = −0.425, *p* = 0.04)
[31]	Retrospective cohort study	To investigate risk factors for CSC unrelated to corticosteroid use.	786 CSC patients (500 males), 3606 controls (2294 male).	/	Exposure to anti-anxiety drugs within a one-year period before enrolment was independently associated with idiopathic CSC among males only (OR, 1.63).
[32]	Retrospective longitudinal study	To investigate risk factors for recurrence in CSC	73 CSC patients	/	Patients with a history of psychiatric illness were associated with an increased risk of CSC recurrence (*p* = 0.007).

## Data Availability

https://pubmed.ncbi.nlm.nih.gov/; info.scopus.com.
